# Study on Acoustic Emission Characteristics of Low-Temperature Asphalt Concrete Cracking Damage

**DOI:** 10.3390/ma14040881

**Published:** 2021-02-12

**Authors:** Kang Yang, Dongxue Li, Zhaoyi He, Hanlin Zhou, Jiaqi Li

**Affiliations:** 1 School of Civil Engineering, Chongqing Jiaotong University, Chongqing 400074, China; zhouhanlin@mails.cqjtu.edu.cn (H.Z.); 622190970103@mails.cqjtu.edu.cn (J.L.); 2 College of Traffic & Transportation, Chongqing Jiaotong University, Chongqing 400074, China; lidongxue@cqjtu.edu.cn (D.L.); hzyzwb@cqjtu.edu.cn (Z.H.)

**Keywords:** asphalt concrete, low temperature, acoustic emission, source location

## Abstract

In this study, asphalt concrete specimens were subjected to a semicircle bending test at −10 °C to simulate the process of the development of cracks in asphalt concrete at low temperature. The acoustic emission parameters were collected during the test, the variation characteristics of acoustic emission parameters were analyzed, and the peakedness value was introduced to evaluate the damage of asphalt concrete. The dynamic evolution of fracture development was analyzed by periods with acoustic emission source location. The results indicate that the damage of asphalt mixtures shows an obvious brittle characteristic at low temperature, acoustic emission signals mainly originate from the crack damage caused by tensile stress, and the strength and number of signals can reflect the degree of crack development. Based on acoustic emission parameters and load curves, the cracking damage of asphalt concrete at low temperature in this study can be divided into three periods: a calm period, a stable development period, and a rapid fracture period. The crack point occurred and propagated upward rapidly in the rapid fracture period. During this period, acoustic emission parameters such as ringing count, acoustic emission energy, and amplitude increased suddenly; furthermore, the peakedness value reached its peak in this period and corresponded well with the low-temperature damage of asphalt concrete. Acoustic emission source location technology can track position of crack points and the propagation path of cracks, reflecting the dynamic evolution process of asphalt concrete crack damage at low temperature.

## 1. Introduction

Asphalt concrete is a typical composite material that is widely used in the construction of road engineering in cold regions. Crack damage of asphalt pavement at low temperature is common; when the internal damage of asphalt concrete reaches a certain extent, micro-cracks will occur and expand, resulting in macro-cracks [1], which seriously affect the pavement performance and traffic safety. However, the unpredictability and seriousness of micro-cracks have always been a major problem in the field of highway engineering.

**Figure 1:**
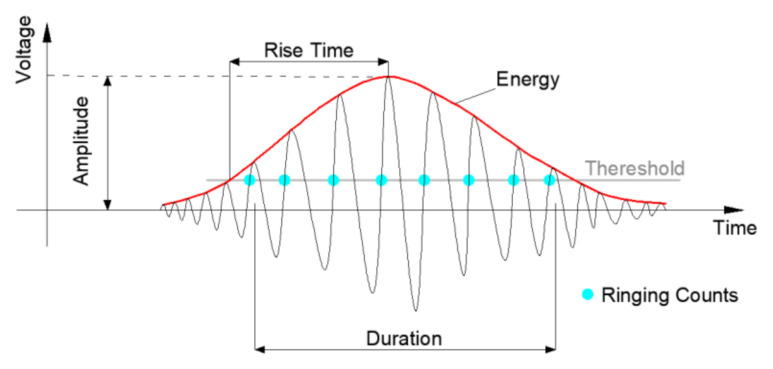
Acoustic emission (AE) parameters.

Acoustic emission (AE) is a nondestructive testing technology that can detect tiny deformations and damage inside materials by using the high-speed digital AE detector and preamplifier; the signals received by sensors are converted and shown by corresponding parameters. In recent years, AE has been widely used in the study of relatively homogeneous brittle materials such as rocks, metal, and concrete [2,3,4,5]. The essence of acoustic emission is the transient elastic wave generated by the rapid release of stress when the material is deformed or damaged [6,7]. By analyzing the characteristics of AE signals, the mechanical and physical behaviors of materials can be evaluated to reflect the subtle changes inside the materials at the microscopic level, which are difficult to reflect from the macroscopic physical parameters such as the stress-strain curve. AE source location technology can also determine the location of damage within the material. Figure 1 presents some basic AE parameters such as amplitude, energy, ringing count, duration, etc.

However, although the application of AE technology to asphalt concrete started late, much attention has been paid to the characterization of the fracture behavior of asphalt concrete through variation in AE parameters and research on the AE source location of fracture points. Jiao et al. [8,9,10] carried out compression and splitting tests on permeable asphalt mixtures, and obtained the evolution law of each AE parameter. The results showed that the variation characteristics of each parameter had a certain relationship with the macro damage of the asphalt mixture, and thus AE technology has great potential in the damage characterization of asphalt concrete. Qiu et al. [11,12] studied the continuous damage evolution behavior of an asphalt mixture and located the damage position using AE location technology. The results indicate that acoustic emission technology can characterize the fracture behavior of asphalt mixtures. Behzad [13] investigated the self-healing ability of an asphalt mixture by AE; the results showed that more cooling cycles could reduce the self-healing ability of the asphalt mixture. AE can be used to analyze the influence of the resting time between cooling cycles on the fracture energy of materials.

AE technology has been gradually applied to the research of the mechanical fracture behavior of asphalt concrete. However, it is worth mentioning that there are still many problems to be solved, such as describing and explaining the relationship between the crack damage of low-temperature asphalt concrete and the characteristics of AE signal, as well as the corresponding relationship between macro crack extension path and AE source location at low temperature. Based on the 16-channel SEAU3H AE equipment manufactured in China, this paper aims to:

(1) Study the variation characteristics and laws of AE parameters during the fracture and damage process of asphalt concrete at −10 °C.
(2) Track the crack propagation path using AE source localization technology and study the dynamic variation characteristics of AE source spatial distribution during the fracture process of asphalt concrete at −10 °C.

## 2. Materials and Methods

### 2.1. Materials

**Figure 2:**
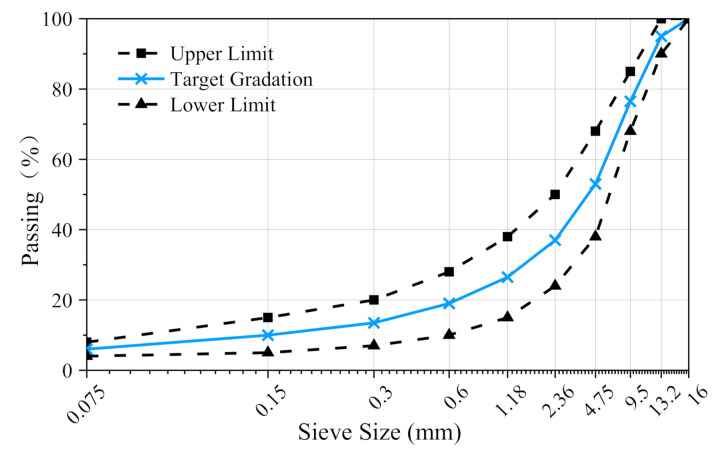
Gradation curve for AC-13.

AC-13 asphalt concrete was utilized with a grade 70 penetration bitumen content of 4.3% and mineral filler content of 6%; the aggregate and mineral fillings were limestone. The target aggregate gradation curve of the asphalt concrete was obtained from the mid-value between the upper and lower limits according to Standard Test Methods of Bitumen and Bituminous Mixtures of Highway Engineering (JTG E20-2011), as shown in Figure 2.

**Figure 3:**
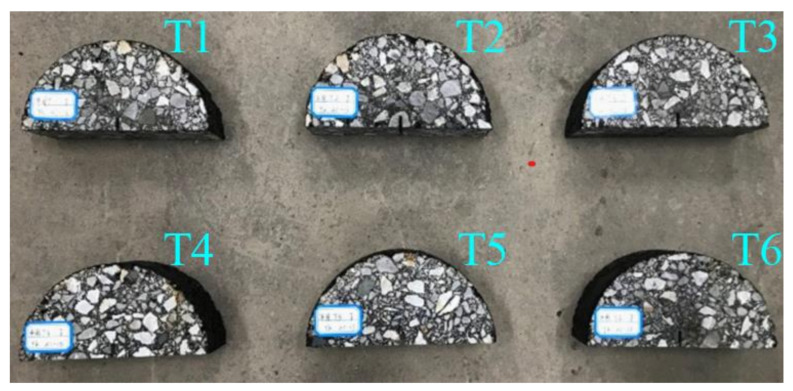
Formed specimens (T1–T6).

After mixing at 160 °C, the AC-13 was compacted by a gyratory compactor at 140 °C to form a cylindrical specimen with a diameter of 150 mm and a height of 180 mm. First, the upper and lower surfaces were cut to 15 mm thick, respectively, and the remaining specimens were cut into three circular specimens with a thickness of 50 mm. Then, six semi-circular specimens with a radius of 75 mm and a height of 50 mm were cut through the central axis. Furthermore, a pre-cut notch of 10 mm in length and 2 mm in width was made at the bottom of the semi-circular specimens, numbered T1–T6. Figure 3 shows the formed specimens:

### 2.2. Test Setup and AE System

A UTM-25 manufactured by SANS in Guangdong China was utilized in the semi-circular bending test; the loading rate was set at 2 mm/min until the end of the test. To ensure the reliability of the test, 6 parallel tests were carried out. Before the test, the specimens were placed in a temperature chamber at −10 °C for more than 12 h.

**Figure 4:**
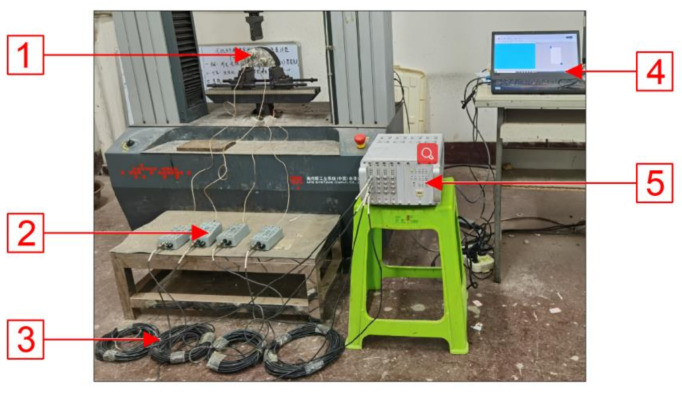
AE test system: 1: G8 sensors; 2: Preamplifier; 3: Cable; 4: Computer; 5: High-speed digital AE detector.

**Figure 5:**
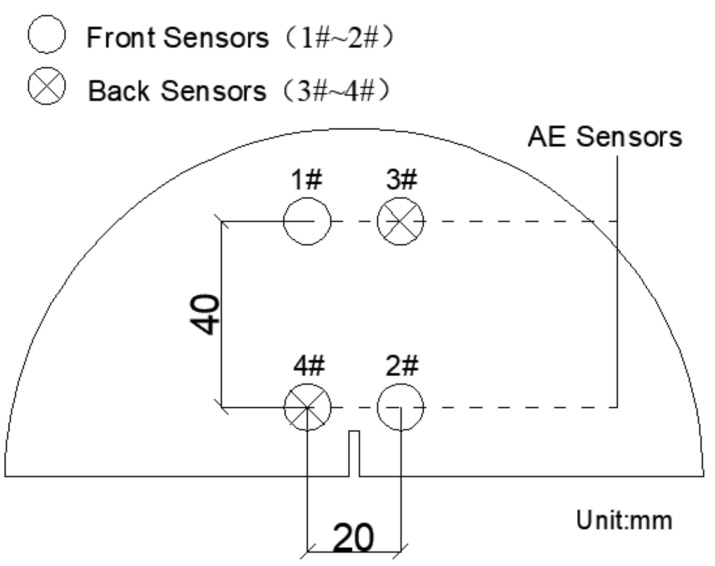
Layout of sensors.

AE tests were simultaneously performed using a 16-channel SEAU3H AE system manufactured by Soundwel in Beijing China. The test equipment is shown in Figure 4. Four AE sensors (G8) were arranged on the surface of the specimen as shown in Figure 5; the receiving frequency of sensors sranged from 18 to180 kHz. The preamplifier was set as 40 dB and the threshold value as 35 dB with a sampling frequency of 1 MHz for the tests.

### 2.3. AE Source Location

**Figure 6:**
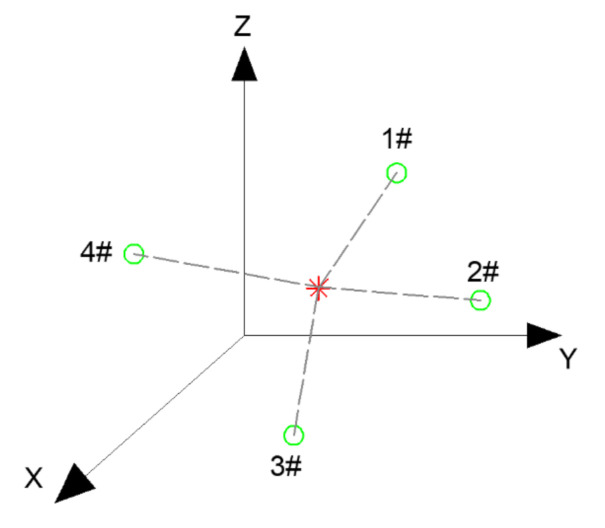
Schematic diagram of time difference location.

AE source location is of great importance in the accurate determination and prediction of damage points. The three-dimensional coordinates of the source points which release elastic waves can be calculated by reasonably arranging the spatial position of the sensors. AE location technology includes time difference and region location, and the time difference location is an accurate and complex location method [14,15,16,17,18,19]. The schematic diagram is shown in Figure 6:

As shown in Figure 6, an AE source was generated at a point inside the material, and the signal propagated to sensors 1# to 4# in turn. The displacement–time relation of signal propagation is as follows:

(1) $${\left(x_{i}-x\right)}^{2}+{\left(y_{i}-y\right)}^{2}+{\left(z_{i}-z\right)}^{2}=v_{p}\times {\left(t_{i}-t_{0}\right)}^{2}$$

where ($x_{i}$, $y_{i}$, $z_{i}$) are the coordinates of the *i*th sensor, $t_{i}$ is the signal arrival time of each sensor, and $v_{p}$ is the propagation velocity inside the asphalt concrete. Furthermore, at least three sensors are required to obtain the 3D coordinates of the AE source. Four sensors were applied in this paper to establish Equation (2):

(2) $${\left(x_{1}-x\right)}^{2}+{\left(y_{1}-y\right)}^{2}+{\left(z_{1}-z\right)}^{2}=v_{p}\times {\left(t_{1}-t_{0}\right)}^{2}\;{\left(x_{2}-x\right)}^{2}+{\left(y_{2}-y\right)}^{2}+{\left(z_{2}-z\right)}^{2}=v_{p}\times {\left(t_{2}-t_{0}\right)}^{2}\;{\left(x_{3}-x\right)}^{2}+{\left(y_{3}-y\right)}^{2}+{\left(z_{3}-z\right)}^{2}=v_{p}\times {\left(t_{3}-t_{0}\right)}^{2}\;{\left(x_{4}-x\right)}^{2}+{\left(y_{4}-y\right)}^{2}+{\left(z_{4}-z\right)}^{2}=v_{p}\times {\left(t_{4}-t_{0}\right)}^{2}$$

By solving Equation (2), the three-dimensional coordinates (x,y, z) and the time ($t_{0}$) of AE source signal generation could be obtained.

## 3. Results and Discussion

AE test data varied for different specimens; however, the variation characteristics and laws of AE parameters were consistent. Therefore, the load data of the representative specimen T2 were selected for discussion and analysis.

### 3.1. Deformation Fracture Characteristics

**Figure 7:**
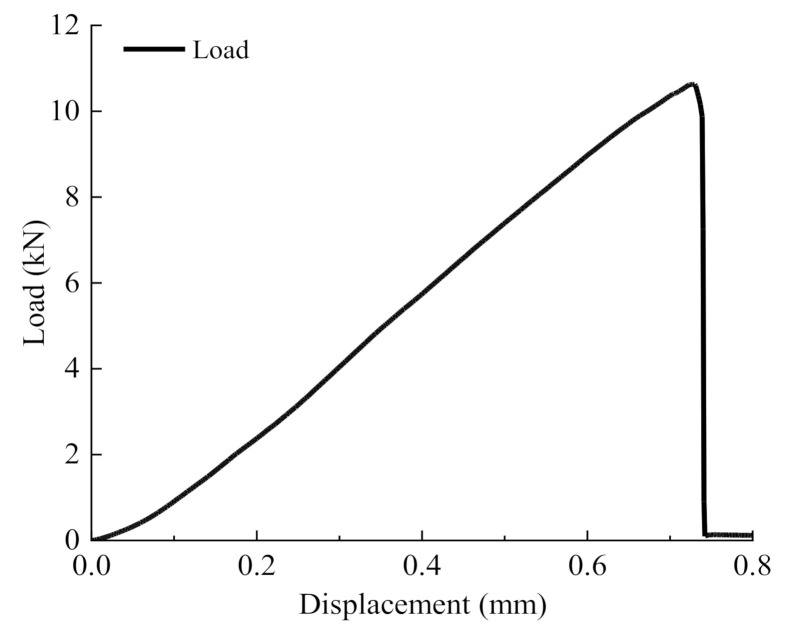
Load–displacement curve.

The load–displacement curve of the semi-circular bending test is shown in Figure 7. According to the curve variation characteristics, the peak load (*Fp*) was about 10.2 kN and the peak vertical deformation was about 0.75 mm. The load had a uniform rate of increase, and increased slowly with the increase of displacement; when the vertical displacement reached about 0.75 mm, the stress decreased instantly, and the specimen was broken.

When the specimen was broken, the peak vertical deformation was about 0.75 mm and no obvious bending deformation occurred. This is an indication that there were some micro-cracks scattered inside the specimen before the bending failure. When the load level reached the peak, the cracking point occurred in the tensile zone and diffused rapidly along the weak zone. Micro-cracks permeated each other in a short time and then developed into the compression zone of the section, and finally formed macroscopic fracture cracks showing obvious brittle fracture characteristics. The main reason for this phenomenon is the high-temperature sensitivity of matrix asphalt.

From the perspective of fracture mechanics, the fracture toughness $K_{II}$ = 0 since the specimen was only under vertical compression, and the pressure was converted to the tension at the pre-cut notch. The equation of fracture toughness $K_{IC}$ can be obtained according to the literature [20]:

(3) $$K_{IC}=\frac{F}{B}f\left(\frac{a}{W}\right)$$

where *F* is the fracture load, which was 10.2 kN in this test; *B* is the thickness of the specimen, which was 50 mm in this test; *a* is the crack length (the pre-cut notch length was 10 mm in this test); and *W* is the strain energy density. According to the literature [20], the shape factor $f\left(\frac{a}{W}\right)$ is as follows:

(4) $$f\left(\frac{a}{W}\right)=0.0684+101.36\left(\frac{a}{W}\right)-3.514{\left(\frac{a}{W}\right)}^{2}+10.53{\left(\frac{a}{W}\right)}^{3}-15.189{\left(\frac{a}{W}\right)}^{4}+11.884{\left(\frac{a}{W}\right)}^{5}$$

According to Equations (3) and (4), $K_{IC}$ is 1.138 MPa·mm^−1/2^, which means that when *K* is greater than 1.138, the specimen will be destroyed; otherwise, it is safe when *K* is less than 2.3.

### 3.2. Characteristics of AE Parameters

Although the specific values of AE parameters were different, the variation trends and rules were the same and consistent. Therefore, the AE data of the representative T2 specimen were selected in this paper for discussion and analysis.

**Figure 8:**
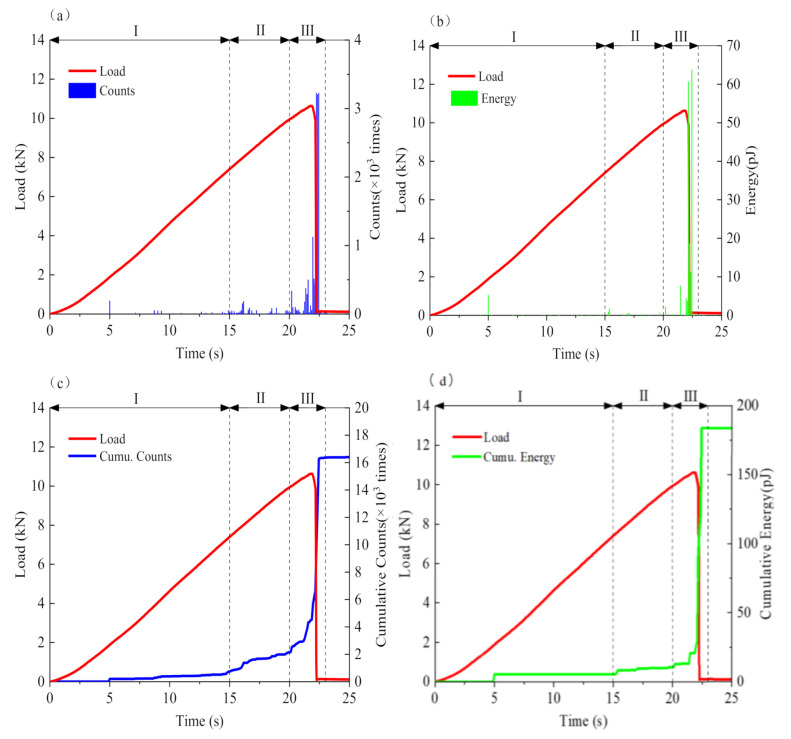

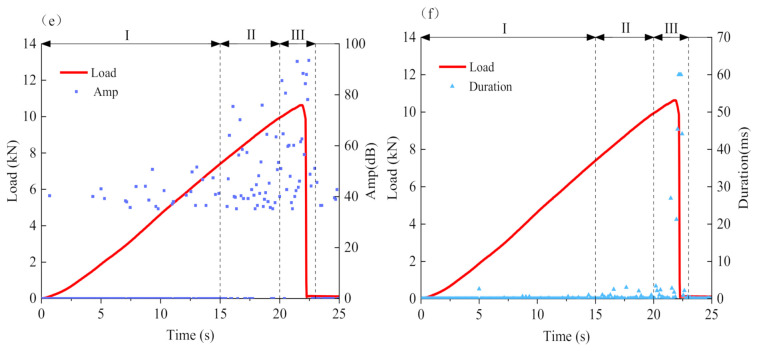
Characteristic AE parameters: (**a**) AE ringing counts; (**b**) AE energy; (**c**) Cumulative ringing counts; (**d**) Cumulative energy; (**e**) Amplitude; (**f**) Duration.

The variation characteristics of AE parameters during the semi-circular bending test are shown in Figure 8. Among them, ringing counts, cumulative ringing counts, and duration can reflect the number of AE activities, while energy, accumulative energy, and amplitude represent the strength of AE activities [21,22,23,24]. As shown in Figure 8, according to the variation characteristics of different parameters, the whole process of asphalt concrete fracture damage at low temperature can be divided into three periods—I: a calm period; II: a stable development period; and III: a rapid fracture period. 

Calm period: During the initial 0–15 s, the load level was 0–0.7 *Fp*. The energy and the ringing counts were low; while there was no obvious upward trend for the cumulative ringing counts and the cumulative energy curve, only sporadic low-strength signals of low amplitude and short duration were generated in the whole period. The main reason is that the specimen itself had tensile and bending strength at low temperature, and the influence of external loads was limited. The stress started to accumulate at the internal weakness points; there was no obvious damage inside the material, and the elastic deformation generated in this period could be recovered, so the AE activity at this period was weak. 

Stable development period: During this period, when the load level was 0.7–0.95 *Fp* and the corresponding time domain was 15–20 s, the ringing counts, energy, and amplitude all increased with the continuous increase of the load level and several longer-duration signals occurred. The cumulative ringing counts and cumulative energy curve had a minor increase at 15 s and then rose steadily. As the influence of external load increased, the stress level at the central section of the specimen began to rise, and the resilience modulus increased. The stress accumulated in the previous period was released locally at the internal weakness, leading to irreversible deformation and damage in the tensile zone, and internal micro-cracks began to occur. Therefore, AE activities were more active than the previous period, while the cumulative ringing counts curve had a higher increase than the cumulative energy curve, indicating that the stress release degree was limited and the micro-cracks only occurred with a local scope. 

Rapid fracture period: In this final period, the load reached peak value and the corresponding time domain was 20–23 s. From 20 s, signal duration was obviously longer and the occurrences of high-level energy, ringing counts, and amplitudes increased in number significantly; the increase rates of cumulative energy and cumulative ringing count curves were accelerated. When the load reached the peak, the main cracks occurred and the AE energy, ringing count, amplitude, and duration all increased to the highest level instantaneously; the cumulative energy and cumulative ringing counts curve rose in a straight line. Subsequently, however, all AE parameters fell to an ultra-low level; the cumulative energy and cumulative ringing counts curves no longer rose. This was mainly due to the increase of vertical deformation at this period, causing the internal micro-cracks begin to extend and interconnect, producing a large number of AE signals of strong penetration. When the load reached the critical stress, the stress in the tensile zone exceeded the ultimate tensile strength; cracks first occurred at the crack point and rapidly propagated to the compression zone, leading to low-temperature brittle fracture. A large amount of energy was released and AE activities were the most active at this time.

It can be seen from Figure 8 that there are obvious relationships between the variation of AE parameters and the deformation and fracture of the asphalt concrete, and AE parameters can reflect the internal microscopic changes that are difficult to detect with load curves. At the initial part of the rapid fracture period, the concentrated and explosive growth of each parameter can be regarded as the forewarning of damage failure of low-temperature asphalt concrete.

### 3.3. Damage Evaluation

**Figure 9:**
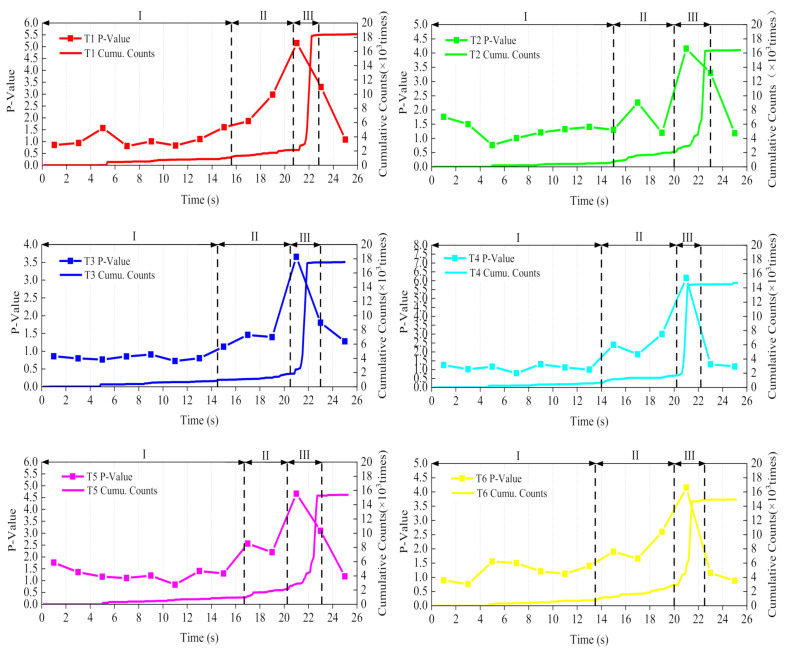
The peakedness (P) variation during the test.

For unstable signals produced by bearing structure vibration, peakedness (P) (K) is often adopted in the monitoring of structure health to evaluate the damage of a bearing structure and its service ability [25,26]. The value of P reflects the irregularity otherness of a set of data; the greater the value of P, the greater the data irregularity and the more unstable the material interior tends to be; conversely, the smaller the value of P, the more stable the material interior tends to be. In this paper, the P value is introduced to evaluate the structural damage of asphalt concrete during the test. The P value is calculated as following:

(5) $$P=\frac{\frac{1}{N}\sum_{i=1}^{N}{\left[x\left(i\right)-\frac{1}{N}\sum_{i=1}^{N}x\left(i\right)\right]}^{4}}{{\left\{\frac{1}{N}\sum_{i=1}^{N}{\left[x\left(i\right)-\frac{1}{N}\sum_{i=1}^{N}x\left(i\right)\right]}^{2}\right\}}^{2}}$$

where P is the kurtosis value, *N* is the number of data samples for each group, and $x\left(i\right)$ is ringing counts in this paper. The ringing counts every two seconds in the test were considered as a set of data, and its P value was calculated, respectively. The results of division of damage periods and P value of six specimens are presented in Figure 9.

As shown in Figure 9, the specific kurtosis value was different for different specimens, while the variation trends and rules were consistent and corresponded to the damage period. For example, taking sample T2 as an example, the P value was small in the initial calm period and ranged from 0.5 to 1.5; in the 16–18 s of the stable development period, the P value suddenly increased to 2.3, the first peak occurred, and then decreased to a lower level; the P value then increased to 4.2 in the 20–22 s of the rapid fracture period; the second peak occurred, which was also the maximum during the whole process; the P value then continued to maintain a higher value of 3.3 in the 22–24 s period; when the specimen was completely fractured, the P value reduced 1.15. 

It can be found from the variation of P that there was a certain correspondence between the value of P and AE activity; the P value was larger when AE activity was more intense and smaller in other stages. P reached its maximum value in the rapid fracture period, indicating that the data irregularity was the greatest at this time, the asphalt concrete specimen approached the ultimate bending strength asymptotically, and was about to be fractured.

### 3.4. AE Source Location

**Figure 10:**
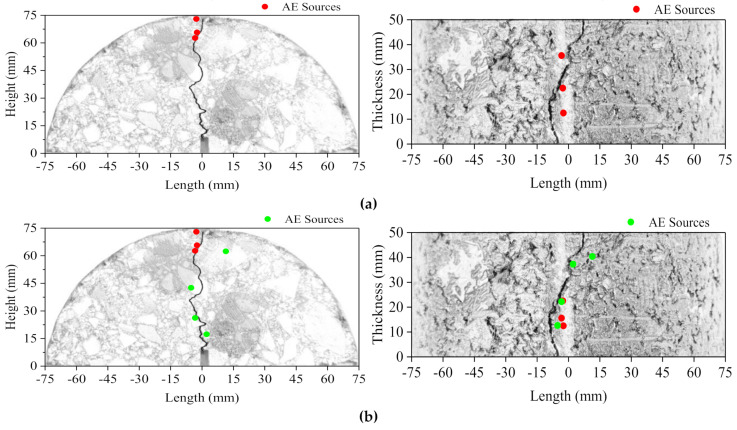

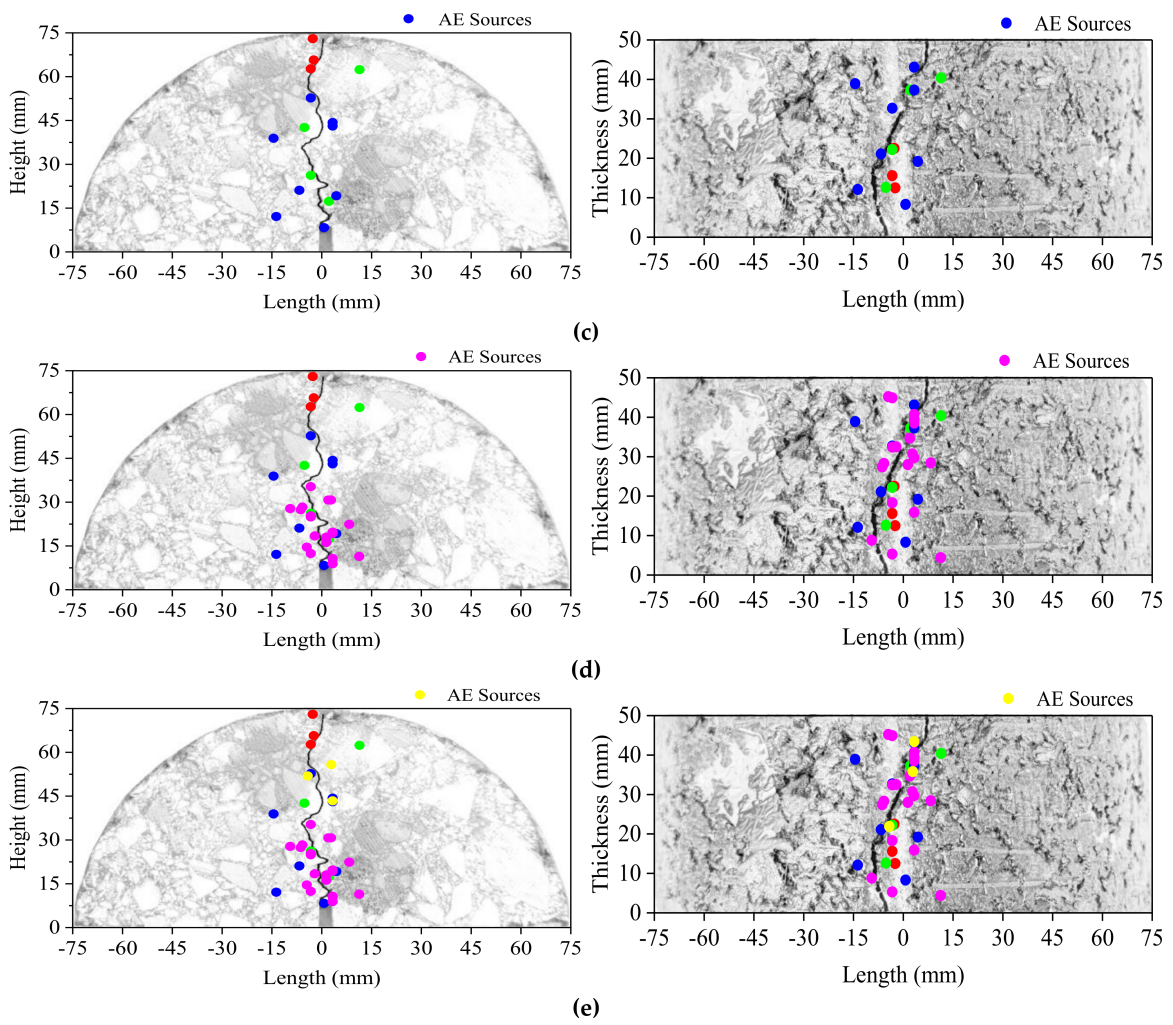
Location of AE sources at each period. (**a**) 0–10 s; (**b**) 10–15 s; (**c**) 15–20 s; (**d**) 20–22 s; (**e**) 22–25 s.

It can be seen from the foregoing that AE parameters have a good ability to reflect cracking damage in low-temperature asphalt concrete. In order to investigate the accuracy of AE source location technology in low-temperature crack propagation, taking sample T2 as an example, planar projection on the front and vertical side was performed on all the three-dimensional locating points obtained in the test, and the results were compared with the actual crack path. The time nodes of source location were selected as 10, 15, 20, 22, and 25 s, and are represented in Figure 10 by different colors, while the solid lines represent the actual crack propagation path. 

Calm period: There were few AE events in this period. Only three AE events occurred at the initial stage (0–10 s), all of which were located in the compression zone of the upper center of the section in the frontside direction, and were distributed in a line in the vertical direction, which was due to the extrusion deformation of the upper part. At the later stage of 10–15 s, four AE Events occurred, one of which was located at the upper part of the section; the other three were distributed at the middle and lower parts of the section in the frontside direction, and were evenly distributed in the vertical direction. AE events decreased in the compression zone and gradually spread to the middle and lower parts, indicating that the AE events mainly resulted in the initiation of a small number of internal micro-cracks caused by tensile stress on specimens.

Stable development period: During this period, AE events increased by eight and mainly scattered in the middle and lower zone, indicating that many internal micro-cracks began to occur in the tensile zone of the section and began to expand in the tensile direction. The scattered distribution of the events in the vertical direction indicates that the internal micro-damage occurred randomly on the vertical section. The growth rate of the number of AE events was increasing; this indicates that the damage process of specimens accelerated with the increase of displacement. 

Rapid fracture period: This period lasted only about 3 s, during which time 20 AE events occurred; this is more than the sum of the previous periods and accounted for 53% of the total number of events. In this period, the micro-cracks rapidly expanded and connected with each other; crack point occurred at the pre-cut notch and propagated upward rapidly, resulting in a macroscopic major crack and causing the specimen to rapidly brittle fracture. AE events in this period all gathered around the crack point and the initial path of propagation in the frontside direction; additionally, the aggregation of AE sources at the center of the vertical direction indicates that the crack was generated at the center of the vertical section. This phenomenon indicates that the occurrence of AE source location has a congruent relationship with the generation of cracks, and the number and density of AE events can reflect the severity of damage, which is forward-looking for the appearance of macroscopic damage regions.

As the displacement continued to increase, the whole section of the specimen was in a state of tension. Finally, three AE events occurred, scattered on the upper part of the section, indicating that the crack continued to propagate upward, and the specimen was eventually completely damaged.

It can be seen, from comparison with the actual cracks, that the location points of AE sources were all gathered around the actual cracks, but did not coincide exactly with them. This is due to the time difference method, which assumes that the propagation of stress wave is stable in an ideal homogeneous material, while the asphalt concrete is a heterogeneous composite material with a large internal irregularity. As a result, the propagation of the stress wave was not in a stable state, but rather always attenuating, thus reducing the accuracy of positioning. However, the error of AE location points and actual cracks did not exceed 1 cm, which meets the engineering requirements, indicating that the positioning result was real and effective and could be used as the engineering basis.

## 4. Conclusions

Based on AC-13 asphalt concrete semicircle bending tests and acoustic emission tests, we analyzed the AE parameters during the cracking process of specimens. The main conclusions are as follows:

(1) The fracture process and load–displacement curve of AC-13 asphalt concrete at low temperature showed obvious brittle fracture characteristics; the specimen fractured at the peak load and the corresponding displacement was about 0.75 mm.
(2) The variation of AE parameters during the fracture process of low-temperature asphalt concrete could be divided into three periods: a calm period, a stable development period, and a rapid fracture period. During the rapid fracture period, AE energy and ringing count increased gradually, and then suddenly, the cumulative energy and cumulative ringing counts reached a mutation point and then rose in a straight line. This variation characteristic can be regarded as a forewarning of macroscopic cracks. The K value reached its maximum at the rapid fracture period and had a good correspondence with the AE parameters, which could be used to evaluate the damage stability in the process of specimen cracking.
(3) The dynamic evolution of the spatial distribution of AE location points can be used to track the surface path of crack development, reflect the initiation and propagation of micro-cracks, and evaluate the whole process of crack development. It has great potential and prospects in the crack detection of asphalt pavement in cold regions.

## Data Availability

The data used to support the findings of this study are included within the article.
